# Overview of chikungunya viral transmission, French Guiana, 2026

**DOI:** 10.2807/1560-7917.ES.2026.31.20.2600296

**Published:** 2026-05-21

**Authors:** Marion Petit Sinturel, Dominique Rousset, Antoine Enfissi, Tsiriniaina Ramavoson, Crepin Kezza, Frédéric Jourdain, Félix Djossou, Tiphanie Succo

**Affiliations:** 1 Santé publique France (French National Public Health Agency), Cayenne - Guyane, France; 2 Centre National de Référence des Arbovirus - Institut Pasteur de Guyane (Arbovirus National Reference Center - French Guiana Pasteur Institute), Cayenne - Guyane, France; 3 Laboratoire de Biologie Médical - Centre Hospitalier de l’Ouest Guyanais, (Medical Biology Laboratory - Saint-Laurent Hospital), Saint-Laurent - Guyane, France; 4 Service d’Accueil des Urgences - Centre Hospitalier de l’Ouest Guyanais, (Emergency Unit - Saint-Laurent Hospital), Saint-Laurent -Guyane, France; 5 Santé publique France (French National Public Health Agency), Saint-Maurice, France; 6 Département des Maladies Infectieuses et Tropicales - Centre Hospitalier de Cayenne (Tropical and Infectious Diseases Unit - Cayenne hospital), Cayenne - Guyane, France

**Keywords:** chikungunya, arbovirus, epidemiology, French Guiana

## Abstract

In January 2026, French Guiana detected its first confirmed chikungunya case, initiating a second outbreak after the first one which occurred in 2014/15. The expected seroprevalence for 2026 is estimated at 16.2%, with the virus likely spreading eastward from the west of French Guiana. The causative East/Central/South African (ECSA) strain, lacking the vector-adaptive E1-A226V mutation, may reduce transmission risk in mainland France and Europe, but Caribbean Islands remain vulnerable due to the vector’s presence.

At the end of 2025 and early 2026, the Pan American Health Organisation (PAHO) reported a sustained increase in chikungunya cases across the Americas, as well as a resumption of autochthonous transmission in areas where the virus had not circulated for several years [1]. Among these countries, Suriname, which shares the Maroni River border with French Guiana, reported its first autochthonous case in late 2025. By 27 February, the Ministry of Health of Suriname reported 1,150 cases and two deaths [2,3]. In French Guiana, the first autochthonous chikungunya case was confirmed by RT-PCR at the end of January (week 4/2026) in the western part of the region, near the Suriname border [4]. This case marked the beginning of the current local chikungunya transmission.

This communication describes the current situation and compares the observed dynamics – the outbreak’s scale, geographical distribution, circulating virus strains and characteristics of hospitalised cases – with those observed in 2014/15.

## Public health response

The regional office of the French National Public Health Agency quickly implemented an epidemiological surveillance system, based on the framework established during the last outbreak of chikungunya in 2014/15. This system relies on three key components: (i) a laboratory-based surveillance system involving all diagnostic laboratories across the territory, supported by the Arbovirus National Reference Center at the French Guiana Pasteur Institute (NRC), which ensures virological confirmation and strain characterisation; (ii) a syndromic surveillance component through a network of general practitioners, emergency department visits at the three regional hospitals, and consultations in decentralised health centres in remote areas, allowing for timely detection of suspected cases; and (iii) the surveillance of hospitalisations and deaths, carried out by public health nurses, to assess the severity and impact of the outbreak.

## Outbreak description

**Figure 1:**
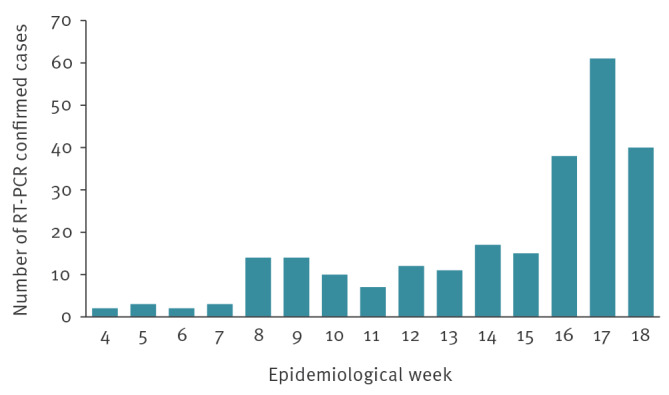
Number of RT-PCR-confirmed chikungunya cases by week, French Guiana, weeks 4/2026–18/2026 (n = 249)

From the first autochthonous chikungunya case identified in January (week 4/2026) to beginning of May (week 18/2026), 249 confirmed chikungunya cases were reported in French Guiana (Figure 1) [5]. The male-to-female ratio was 0.7, and the median age was 29 years (interquartile range (IQR): 12–48), with 74 (30%) of cases aged 3–14 years. The male-to-female ratio aligns with findings from the 2014/15 chikungunya outbreak analysis (sex ratio M/F: 0.7) [6] and with a seroprevalence study which reported a seroprevalence of 22% in females compared with 18.5% in males [7].

## Geographical distribution

**Figure 2:**
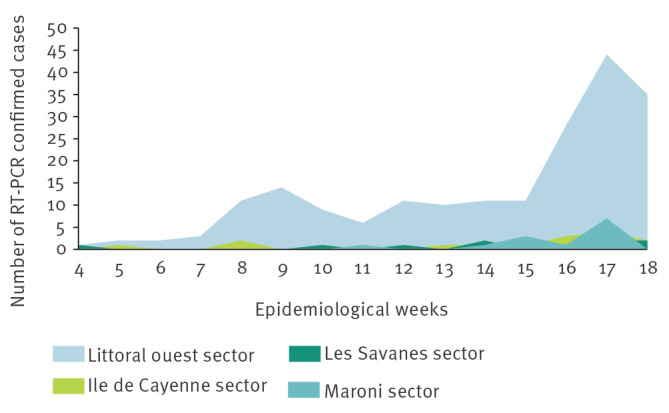
Geographical distribution of RT-PCR-confirmed chikungunya cases by weeks, French Guiana, weeks 4/2026–18/2026 (n = 236)

Place of residence within French Guiana was known for 236 cases. Geographically, from January (week 4/2026) to May (week 18/2026), most cases (n = 198; 80%) were concentrated in the western part of the region*,* near the Suriname border (Figure 2). Among cases reported outside this location, 15 were located in Ile de Cayenne sector, 10 in Les Savanes sector and 13 in Maroni sector. An increasing trend is observed in Maroni sector.

## Hospitalisations

**Figure 3:**
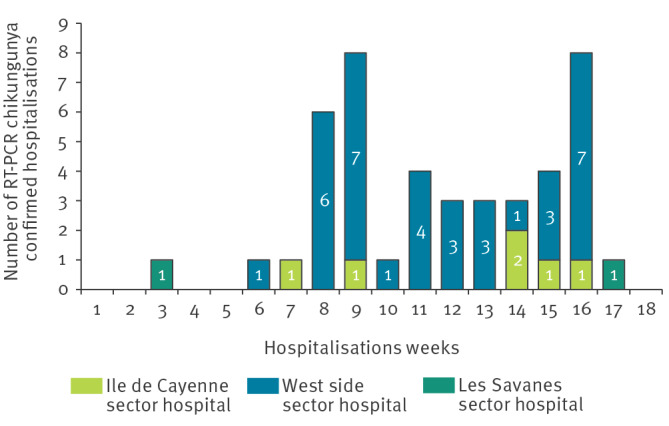
Geographical distribution of confirmed and probable chikungunya hospitalisations by hospital location and by week, French Guiana, weeks 4/2026–18/2026 (n = 44)

Since the start of the virus circulation, 44 hospitalisations have been recorded across the three hospitals of French Guiana (Figure 3), with 36 hospitalised in the in the western part of French Guiana, where the virus is mostly circulating. Among the 44 hospitalised cases, the male-to-female ratio was 0.7, the median age was 28 years (IQR: 10–56), and the median hospitalisation duration was 52 h (IQR: 37–92).

Of these hospitalisations, 31 cases were classified as 'usual cases', 11 as 'unusual cases' (hospitalised cases with unusual non-severe multisystem involvement [6]) and two as a 'severe case' (organ failure); the final classification of 20 of these confirmed and probable 44 cases remain to be validated by an infectiologist. Among hospitalised cases, 25 had risk factors and/or comorbidities, including arterial hypertension, obesity, diabetes, pregnancy or others. One death has been reported among confirmed cases who were hospitalised; infectious disease specialists are still assessing the direct link between the death and infection with the chikungunya virus.

## Phylogenetic analysis

From a virological perspective, the NRC characterised the strains by sequencing two isolates (GenBank accession numbers: PZ246469 - 70) from the first confirmed cases identified in French Guiana: one was autochthonous and the other was an imported case from Suriname.

**Figure 4:**
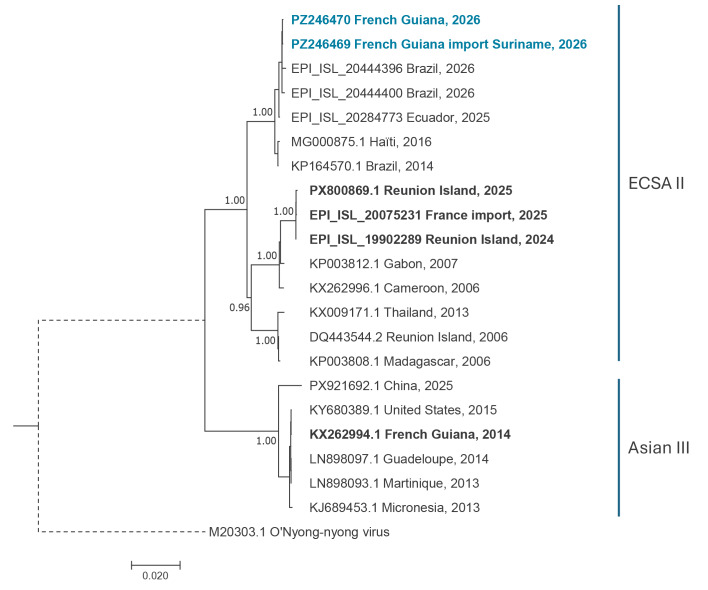
Phylogenetic tree of chikungunya virus isolated from the outbreak cases, French Guiana, weeks 4/2026–5/2026 (n = 2)

The tree was constructed using the neighbour-joining method based on an 11,175 bp fragment. Taxon labels indicate the strain designation, country and year of isolation. Nodes with posterior probabilities (clade credibilities) > 0.95 have been labelled. The authors gratefully acknowledge the originating and submitting laboratories who submitted the sequences used in the phylogenetic analysis to NCBI and GISAID and extend special thanks to the laboratories that provided the most recent South American sequences: Instituto Butantan, Sao Paulo, Brazil and Centro de Referencia Nacional de Genomica, Secuenciacion y Bioinformatica GENSBIO, Quito, Ecuador.

## Discussion

Chikungunya is an infectious disease caused by an arbovirus of the genus *Alphavirus*, transmitted to humans by mosquitoes of the genus *Aedes*. In French Guiana, the main vector is *Aedes aegypti*, unlike in Europe, where *Aedes albopictus* is the species responsible for transmitting the virus.

The chikungunya outbreak in 2014/15 lasted 20 months and resulted in 16,000 suspected cases, with a cumulative incidence of 62 cases per 1,000 inhabitants, 500 hospitalisations and one death [6]. In 2017, the Pasteur Institute estimated the chikungunya seroprevalence within the French Guiana population at 20.3%, 2 years after the end of the outbreak [7]. At 1 January 2025, the French Guiana population was estimated at 292,354 inhabitants [8], with 20% (58,471 individuals) younger than 10 years. This younger population was not exposed to the 2014/15 outbreak and can be considered as immunologically naïve to chikungunya. Consequently, the expected global seroprevalence in French Guiana in 2026 is estimated at 16.2%. The feasibility of modelling the number of future hospitalisations using data from the last outbreak is currently assessed.

In addition, during the 2014/15 outbreak, the age group 0–14 years represented a proportion of chikungunya cases (25%) comparable to 2026, but the age groups 45–59 and ≥ 60 years had the highest incidence rates, each at 4.3% (vs 2.2% for the 0–14-year-olds) [6]. The seroprevalence study found no significant age-related differences, suggesting the same risk of infection for all age categories [7].

At the beginning of 2026, the outbreak’s onset was most probably linked to the ongoing epidemic in Suriname. The Maroni River is indeed more a transboundary living area than a closed border, with frequent population movements across the river [9], increasing the risk of disease transmission between the countries. Currently (May 2026), the virus is expected to spread eastward within French Guiana, although no cases have so far been confirmed in the eastern region of the territory or in the Amapà region of Brazil (Monthly transboundary meetings: SVS Amapá, Brazilian Ministry of Health, April 2026).

The high number of hospitalisations may be due to the method used to calculate length of stay, which relies on medical records timestamps. This approach may lead to misclassification, as it does not distinguish between stays shorter than 24 h which should not be counted as hospitalisations, and those exceeding this threshold. Among the 44 cases considered hospitalised for over 24 h, 20 were treated in emergency departments. In addition, during the early phase of an epidemic, physicians tend to monitor patients more closely, especially those with comorbidities or limited access to care. These factors, combined with short hospitalisation durations and only one severe case, suggest an overestimation of the hospitalisation proportion.

Phylogenetic analysis done by NRC revealed high genetic similarity, and a close relationship with recent sequences from Ecuador and Brazil [1]. The strain belongs to the East/Central/South African (ECSA) lineage, unlike the Asian lineage strain responsible for the 2014/15 outbreak in French Guiana [10]. Given that Brazil already documented ECSA circulation in 2014/15 [11], and that this lineage has persisted in the Americas since then, the currently circulating ECSA strain probably derives from it. Furthermore, the current ECSA strain differs from those responsible for earlier outbreaks in Réunion Island [12] and mainland France [13]. It lacks, notably, the E1-A226V mutation, which is associated with enhanced adaptation to *Aedes albopictus* – a feature that may limit, but does not preclude, the risk of transmission in mainland France and Europe [14]. Frequent travel and close connections with other territories in the Americas – particularly the Caribbean Islands – could also facilitate regional spread, indicating that continued surveillance is needed. Although the 2014/15 strain in French Guiana differs from the current one, individuals who have acquired natural immunity following infection [15] in 2014 and 2015 remain protected for the strain circulating in 2026. 

## Conclusion

The dynamic of the 2014/15 outbreak, with a high number of cases and hospitalisations, the low immunity in the population in 2026, the presence of the vector, and the current rainy season—which is conducive to vector proliferation—are factors that contribute to viral transmission within French Guiana. At present, control measures focus on vector control targeting both adult mosquitoes and larvae in and around areas of virus circulation as well as on community engagement. The deployment of innovative vector control methods, such as *Wolbachia*-based strategies, could also be explored for future implementation.

## Data Availability

All epidemiological data presented in the manuscript are available from the corresponding author upon reasonable request. Sequencing data of the French Guiana strains from NRC have been deposited in the GenBank database under the accession number PZ246469 – 70.

## References

[1] Pan American Health Organization (PAHO). Epidemiological alert – Chikungunya. Washington, D.C.: PAHO; 10 Feb 2026. Available from: https://www.paho.org/sites/default/files/2026/02/2026-february-10-phe-alert-chkv-finalen.pdf

[2] Suriname Ministry of Health. Current figures on chikungunya – Bilthoven: National Institute for Public Health and the Environment; 27/02/2026. Available from: https://www.rivm.nl/en/chikungunya/current-figures

[3] StartNieuws. Al 1150 chikungunya besmettingen, tweede sterfgeval onderzocht. [Already 1,150 chikungunya infections, second death investigated]. Paramaribo: StartNieuws; 25 Feb 2026. Dutch. Available from: https://www.starnieuws.com/index.php/welcome/index/nieuwsitem/90778

[4] Carvalho L, Devos S, Petit-Sinturel M, Succo T. Bulletin bi-mensuel de surveillance épidémiologique. Région Guyane. Semaines 04 et 05 (du 19 janvier au 1er février 2026). [Bi-monthly epidemiological surveillance bulletin. French Guiana region. Weeks 4 and 5 (19 Jan to 1 Feb 2026)]. Saint-Maurice: Santé publique France; 2026. French. Available from: https://www.santepubliquefrance.fr/sites/default/files/rdd/document/bullreg_guyane_20260205.pdf

[5] Bulletin de surveillance épidémiologique. Région Guyane. Semaine 13 (du 23 au 29 mars 2026). [Epidemiological surveillance bulletin. French Guiana region. Week 13 (March 23-29, 2026)]. Saint-Maurice: Santé publique France; 2026. French. Available from: https://www.santepubliquefrance.fr/sites/default/files/rdd/document/bullreg_chik_guyane_20260402.pdf

[6] Petit-Sinturel M, Carvalho L, Succo T. Bulletin thématique. Bilan de l’épidémie de chikungunya 2014-2015 en Guyane: Une maladie en émergence. Mars 2026. [Thematic bulletin. Review of the 2014-2015 chikungunya epidemic in French Guiana: An emerging disease. March 2026]. Saint-Maurice: Santé publique France; 2026. French. Available from: https://www.santepubliquefrance.fr/sites/default/files/rdd/document/bullreg_chik_guyane_bilan_2014_2015.pdf

[7] Bailly S Rousset D Fritzell C Hozé N Ben Achour S Berthelot L Spatial distribution and burden of emerging arboviruses in French Guiana. Viruses. 2021;13(7):1299. 10.3390/v13071299 34372505 PMC8310293

[8] Lépine F-X, Mocquet C. Le dynamisme démographique faiblit avec des naissances moins nombreuses - Bilan démographique 2024 en Guyane. [Demographic growth is slowing with fewer births - 2024 demographic overview in French Guiana]. Montrouge: National Institute of Statistics (INSEE); 2025. French. Available from: https://www.insee.fr/fr/statistiques/8556999

[9] Léobal C. « Manger des deux pays »: habiter le fleuve Maroni, frontière amazonienne de l’Europe (Guyane/Suriname). [“Eating from both countries”: living on the Maroni River, the Amazonian border of Europe (French Guiana/Suriname)]. Revue européenne des migrations internationales. 2024;40(1):171-92. French. Available from: https://journals.openedition.org/remi/25662

[10] Leparc-Goffart I Nougairede A Cassadou S Prat C de Lamballerie X. Chikungunya in the Americas. Lancet. 2014;383(9916):514. 10.3390/genes15111365 24506907

[11] Fritsch H Giovanetti M Clemente LG da Rocha Fernandes G Fonseca V de Lima MM Unraveling the complexity of chikungunya virus infection immunological and genetic insights in acute and chronic patients. Genes (Basel). 2024;15(11):1365. 10.2807/1560-7917.ES.2025.30.22.2500344 39596565 PMC11593632

[12] Frumence E Piorkowski G Traversier N Amaral R Vincent M Mercier A Genomic insights into the re-emergence of chikungunya virus on Réunion Island, France, 2024 to 2025. Euro Surveill. 2025;30(22):2500344. 10.2807/1560-7917.ES.2025.30.22.2500344 40476290 PMC12143119

[13] Frumence E Piorkowski G Traversier N Amaral R Vincent M Mercier A Genomic insights into the re-emergence of chikungunya virus on Réunion Island, France, 2024 to 2025. Euro Surveill. 2025;30(22):2500344. 10.2807/1560-7917.ES.2025.30.22.2500344 40476290 PMC12143119

[14] Fortuna C Toma L Remoli ME Amendola A Severini F Boccolini D Vector competence of Aedes albopictus for the Indian Ocean lineage (IOL) chikungunya viruses of the 2007 and 2017 outbreaks in Italy: a comparison between strains with and without the E1:A226V mutation. Euro Surveill. 2018;23(22):1800246. 10.2807/1560-7917.ES.2018.23.22.1800246 29871722 PMC6152176

[15] Auerswald H Boussioux C In S Mao S Ong S Huy R Broad and long-lasting immune protection against various Chikungunya genotypes demonstrated by participants in a cross-sectional study in a Cambodian rural community. Emerg Microbes Infect. 2018;7(1):13. 10.1038/s41426-017-0010-0 29410416 PMC5837154

